# Knockdown of EpCAM Enhances the Chemosensitivity of Breast Cancer Cells to 5-fluorouracil by Downregulating the Antiapoptotic Factor Bcl-2

**DOI:** 10.1371/journal.pone.0102590

**Published:** 2014-07-14

**Authors:** Jiujiao Gao, Qiu Yan, Shuai Liu, Xuesong Yang

**Affiliations:** Department of Biochemistry and Molecular Biology, Dalian Medical University, Liaoning Provincial Core Lab of Glycobiology and Glycoengineering, Dalian, People’s Republic of China; University of Navarra, Spain

## Abstract

Resistance to fluoropyrimidine-based chemotherapy is the main reason for the failure of cancer treatment, and drug resistance is associated with an inability of tumor cells to undergo apoptosis in response to treatment. Alterations in the expression of epithelial cell adhesion molecule (EpCAM) affect the sensitivity or resistance of tumor cells to anticancer treatment and the activity of intracellular signaling pathways. However, the role of EpCAM in the induction of apoptosis in breast cancer cells remains unclear. Here, we investigated the effect of EpCAM gene knockdown on chemosensitivity to 5-fluorouracil (5-FU) in MCF-7 cells and explored the underlying mechanisms. Our results showed that knockdown of EpCAM promoted apoptosis, inhibited cell proliferation and caused cell-cycle arrest. EpCAM knockdown enhanced the cytotoxic effect of 5-FU, promoting apoptosis by downregulating the expression of the anti-apoptotic protein Bcl-2 and upregulating the expression of the pro-apoptotic proteins Bax, and caspase3 via the ERK1/2 and JNK MAPK signaling pathways in MCF-7 cells. These results indicate that knockdown of EpCAM may have a tumor suppressor effect and suggest EpCAM as a potential target for the treatment of breast cancer.

## Introduction

Breast cancer is currently the most frequently diagnosed cancer and the leading cause of cancer-related death in women worldwide, accounting for 23% of cancer diagnoses and 14% of cancer deaths each year [1]. Therefore, the development of effective therapies against cancer is important. Combination therapy with chemotherapeutic agents such as 5-fluorouracil (5-FU), epirubicin and cyclophosphamide (FEC) is effective to enhance the antitumor effect of inhibitors in early-stage breast cancer [2], [3]. Russo et al. showed that certain proteins such as zonulin, glucagon-like peptide-2 (GLP-2), epidermal growth factor (EGF) and ghrelin play a role in the response to FEC in breast cancer cells [4]. Previous studies have shown that the high mortality of breast cancer can be partly attributed to the acquisition of drug resistance during chemotherapy [5], [6]. Despite the steady improvement of 5-FU-basedtreatment regimens, the patient response rate to 5-FU-based chemotherapy remains modest mainly due to the development of drug resistance. Acquired resistance to 5-FU is a serious therapeutic obstacle to the treatment of breast cancer patients. One major resistance mechanism utilized by tumor cells is to resist drug-induced cell death through the disruption of apoptotic pathways. Therefore, there is an urgent need to develop chemosensitizers capable of increasing the sensitivity of tumor cells to chemotherapy. For this purpose, it is essential to understand the mechanisms of drug resistance and to discover novel strategies to further improve the effectiveness of 5-FU.

Epithelial cell adhesion molecule (EpCAM) is a membrane glycoprotein that is expressed in a subset of normal epithelia and is highly expressed on most carcinomas, including breast cancer. EpCAM therefore has potential as a diagnostic and prognostic marker for a variety of carcinomas [7], [8]. EpCAM is frequently overexpressed in human invasive breast cancer [9]. In our previous study, we found that EpCAM promoted EMT in breast cancer cells. Recent increasing evidence suggests that EpCAM plays an important role in prostate cancer cell proliferation, invasion, metastasis and chemo/radio resistance associated with the activation of the PI3K/Akt/mTOR signaling pathway. Therefore, EpCAM is a novel therapeutic target to sensitize prostate cancer cells to chemo/radiotherapy [10]. EpCAM regulated lung cancer lymph node metastasis in endobronchial ultrasound-guided transbronchial aspiration samples [11]. Although a previous study demonstrated that EpCAM knockdown is effective in the prevention of breast cancer invasion and metastasis, the direct cytotoxicity of EpCAM in breast cancer and the underlying mechanisms remain unclear. The ability of tumor cells to escape from apoptosis is complex. One of the major contributing factors is the elevated level of the anti-apoptotic protein B-cell lymphoma 2 (Bcl-2), which is a key regulator of the mitochondrial pathway of apoptosis [12], [13], [14]. Deregulation of the Bcl-2 protein plays a major role in tumor formation and in the cellular responses to anticancer therapy [15].

In the present study, we investigated the effect of EpCAM on the chemosensitivity of breast cancer cells. Our results showed that knockdown of EpCAM enhances the chemosensitivity of breast cancer cells to 5-FU by downregulating the expression of Bcl-2, suggesting EpCAM as a promising target for anti-cancer therapy.

## Materials and Methods

### Reagents

MCF-7 cells were obtained from the American Type Culture Collection (ATCC). Lipofectamine 2000 Reagent was purchased from Invitrogen (Carlsbad, California, USA). 5-FU and DAPI were purchased from Sigma (St. Louis, MO, USA). Anti-Bcl-2, anti-Bax, anti-Caspase 3, anti-GAPDH were obtained from Santa Cruz. Anti-ERK and anti-p-ERK, anti-JNK and anti-p-JNK were obtained from Cell Signaling (Boston, MA, USA). Cell Counting Kit-8 (CCK8) was purchased from Bytotime Company (Nantong, Jiangsu Province, China). Annexin V/FITC kit was purchased from KeyGEN BioTECH (Nanjing, Jiangsu Province, China). Chemiluminesence (ECL) assay kit was purchased from Amersham (Arlington Heights, USA).

### Cell culture

Human breast cancer MCF-7 cells was maintained in medium DMEM supplemented with 10% calf serum, 1% Pen/Strep, 1 mM sodium pyruvate, 1.5 g/L sodium bicarbonate, and 10 mM HEPES. Cells were incubated in a 5% CO_2_ humidified atmosphere at 37°C.

### Cell viability assay

Cells (2×10^3^/100 µl) were seeded in 96-well plates and treated on the following day with 5-FU, si-EpCAM or si-EpCAM in combination with 5-FU. Cell viability was analyzed using CCK8 kit according to the manufacturer’s instructions, and optical density (OD) was read at 450 nm on a microplate reader (Bio-Rad, California, USA). The Viability (%) was calculated according to the following equation: Viability (%) = (OD treated/OD medium)×100%. The inhibition rate was calculated according to the following equation: Inhibition rate (%) = (1−OD treated/OD control)×100%.

### Cell morphology

Cells (1×10^5^/2 ml) were seeded in 6-well plates and grown for 24 h in order to attach to the surface of the plates completely. They were treated with 5-FU or si-EpCAM in combination with 5-FU. After incubation for another 48 hr, cell morphology was photographed by the inverted green light microscope (Olympus, Tokyo, Japan).

### Detection of apoptosis by DAPI staining

Morphological evaluation of cell apoptosis was performed using DAPI staining which detected the nuclei of both apoptotic and living cells. Cells grown on the glass cover slips were fixed with 4% paraformal dehyde/PBS for 30 min, washed for 15 min in 0.1% Triton X-100/PBS, and incubated in dark with DAPI (10 mg/ml) for 15 min. The stained cells were studied using a fluorescence microscope. The rate of apoptotic cells was recorded in 10 random nonoverlapping fields by two blinded observers. Stained nuclei were visualized under UV excitation and photographed using an Olympus fluorescence microscopy (Olympus, Tokyo, Japan).

### Apoptosis assay by AnnexinV-FITC/PI staining

Cells (1×10^5^/2 ml) were seeded in 6-well plates and treated on the following day with 5-FU, si-EpCAM or si-EpCAM in combination with 5-FU. After incubation for another 48 hr, adherent and floating cells were harvested, washed with PBS, and stained using an Annexin V-FITC/PI kit according to the manufacturer’s instructions. Apoptosis was then analyzed by a FACScan flow cytometer of 20,000 cells in each group. Data analysis was performed with the standard Cell Quest Software. All experiments were performed in duplicate and reproducibility was checked in three independent experiments.

### Cell-cycle phase distribution assay

Cells (1×10^5^/2 ml) were seeded in 6-well plates and treated on the following day with EpCAM plasmid, 5-FU or EpCAM plasmid in combination with 5-FU. After incubation for another 48 hr, non-adherent cells were removed by washing with PBS. Cells were trypsinized, collected and washed twice with PBS. Cell pellets were resuspended in 0.5 ml of PBS and fixed in 4.5 ml of 70% ethanol overnight. Cells were collected by centrifugation and the pellets were resuspended in 0.2 mg/ml of PI containing 0.1% Triton X-100 and 0.1 mg/ml RNase A. The cell suspension was incubated in the dark for 30 min at room temperature and subsequently analyzed on FACScan flow cytometer for DNA content. The percentages of cells in different phases of the cell cycle were sorted using a ModFit 5.2 computer program. The percentages of cells at the G0/G1, S, and G2/M phases were obtained from three independent experiments.

### siRNA knockdown analysis

RNAi-mediated knockdown was performed with the following short interfering RNA (siRNA): EpCAM-1: 5′-UGCUCUGAGCGAGUGAGAATT-3′; EpCAM-2: 5′-UUCUCACUCGCUCAGAGCATT-3′, Negative control siRNA was used in each experiment as a non-silencing control siRNA (siRNA). All siRNAs (20 nM) targeting EpCAM was introduced in cells using lipofectamin 2000 reagent according to the manufacturer’s protocol.

### Western blot

To prepare whole cell extracts, cells at 90% confluent were washed in phosphate-buffered saline (PBS) before incubation with lysis buffer (1% Triton X-100, 150 mM NaCl, 10 mM Tris, pH 7.4, 1 mM EDTA, 1 mM EGTA, pH 8.0, 0.2 mM Na_3_VO_4_, 0.2 mM phenylmethylsulfonyl fluoride, 0.5% Nonidet P-40) on ice for 10 min. The cell lysates were clarified by centrifugation at 9000×g for 10 min and the supernatants were collected. Protein concentration was determined with the Coomassie Protein Assay Reagent using bovine serum albumin (BSA) as a standard. Cell lysates (50 µg) were separated by 10% SDS-PAGE min-gel. Samples were transferred electrophoretically to nitrocellulose membranes, blocked with TTBS containing 5% fat-free dry milk for 2 h and incubated for 3 h with the appropriate primary antibodies at the dilutions recommended by the suppliers. After incubation with a HRP-conjugated anti-goat secondary antibody, immunoreactive proteins were visualized with ECL detection system. Western blots shown are representative of at least three independent experiments. Densitometry of each band for the target protein was quantified by densitometry analysis with Labworks 4.6. The protein band intensity was quantified by the mean±SEM of three experiments for each group as determined from densitometry relative to β-actin.

### Statistics

The quantitative data derived from three in dependent experiments are expressed as means (±S.D.). Unpaired Student’s t-tests were used to analyze between group differences that is repeated and P-value<0.05 was considered statistically significant.

## Results

### Knockdown of EpCAM in combination with 5-FU decreases the viability of breast cancer cells

**Figure 1:**
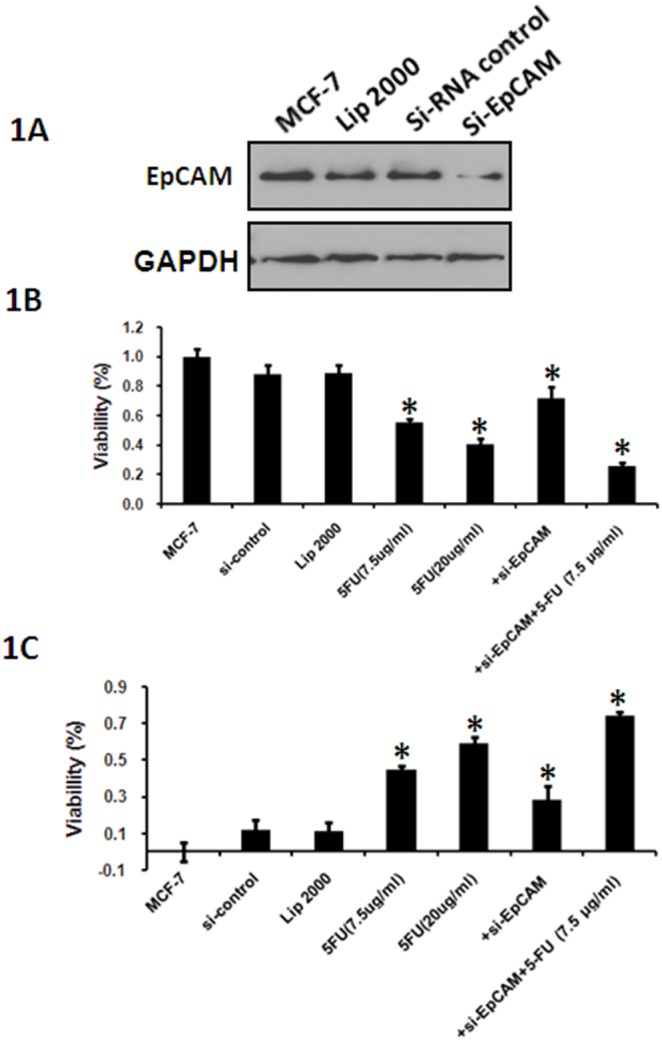
Effect of si-EpCAM and/or 5-FU treatment on cell viability in vitro. MCF-7 cells were treated with 5-FU (7.5 µg/ml and 20 µg/ml) and/or si-EpCAM. A. MCF-7 cells were treated with si-EpCAM, the expression of EpCAM was detected. A negative siRNA control and Lipofectamin 2000 were served as control. B. Cell viability was detected with the CCK-8 assay. Cell viability was expressed as a percentage of control cells (MCF-7 cells). B. The results are presented as the inhibitory ratio of MCF-7 cells. *P<0.05.

First we detected the EpCAM expression after treated with si-EpCAM, and the result showed that EpCAM expression decreased (Fig. 1A). To evaluate the cytotoxicity of 5-FU and the effect of small interfering RNA mediated silencing of EpCAM (si-EpCAM), MCF-7 cells were incubated with 7.5 µg/ml and 20 µg/ml 5-FU combined with or without si-EpCAM for 48 h. Cell viability was determined using the CCK-8 assay kit. The results showed that 5-FU significantly decreased the viability of MCF-7 cells in a dose-dependent manner (Fig. 1B, columns 1, 2, 3), indicating that MCF-7 cells are sensitive to 5-FU. Treatment with si-EpCAM in combination with 5-FU further decreased cell viability compared with 5-FU or si-EpCAM treatment alone (Fig. 1B).

To evaluate the inhibitory effect of 5-FU treatment on MCF-7 cells, the proliferation of cells treated with 5-FU at 7.5 µg/ml and 20 µg/ml was detected using the CCK-8 assay after incubation for 48 h. The results showed that 5-FU alone inhibited the proliferation of MCF-7 cells in dose-dependent manner (Fig. 1C, columns1, 2, 3). To evaluate the synergistic effect of si-EpCAM and 5-FU, MCF-7 cells were treated with si-EpCAM combined with or without 5-FU and cell viability was assessed using the CCK-8 assay. As shown in Fig. 1C, treatment with si-EpCAM in combination with 5-FU increased the inhibitory effect compared with 5-FU or si-EpCAM treatment alone, further supporting the synergistic effect.

### Knockdown of EpCAM in combination with 5-FU affects the morphology of breast cancer cells

**Figure 2:**
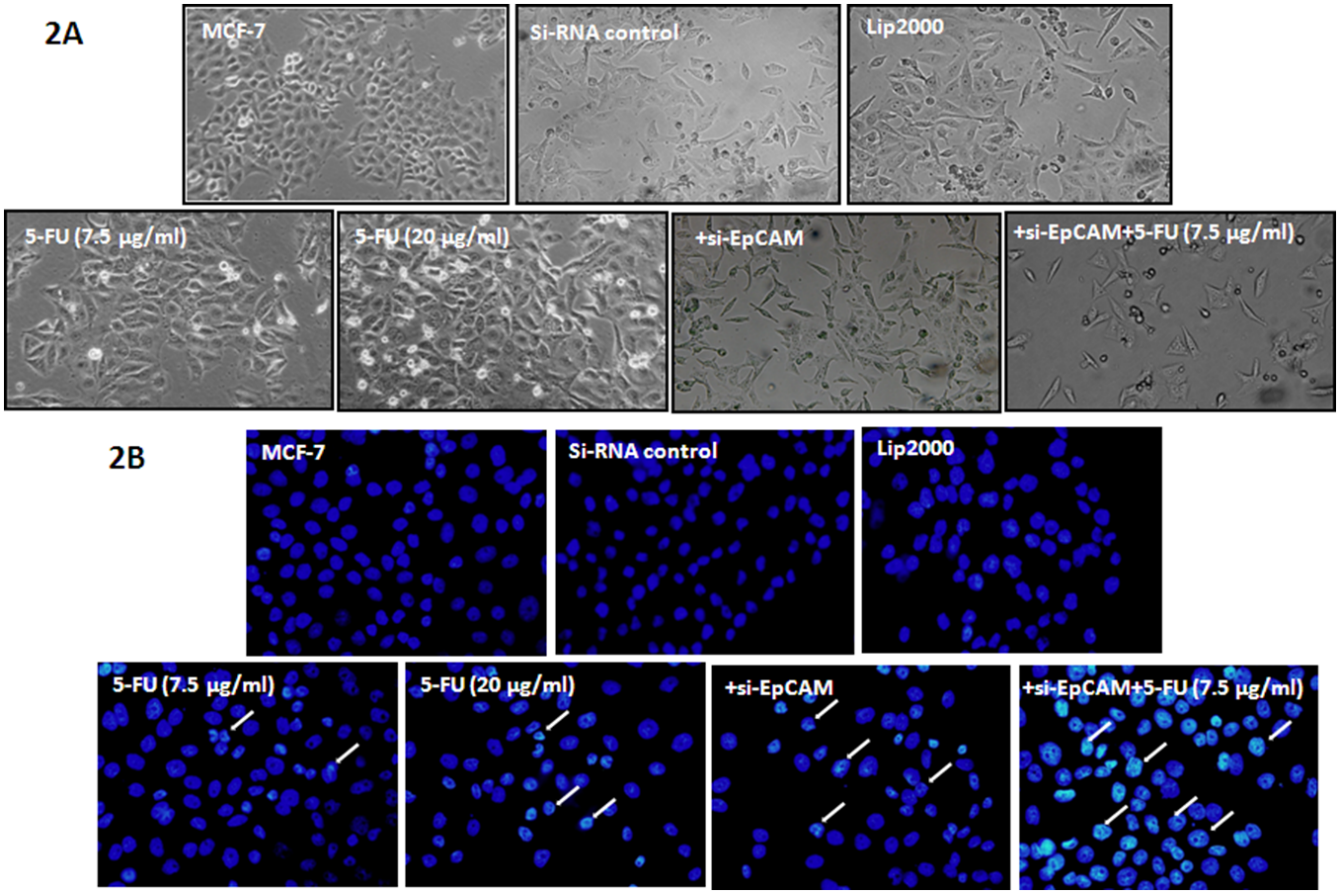
Effect of EpCAM silencing and/or 5-FU treatment on the morphology of MCF-7 cells. A. Morphologic changes of MCF-7 cells treated with the indicated concentration of si-EpCAM and 5-FU alone or together for 48 h. Magnification: 100×. B. MCF-7 cells were treated with the indicated concentrations of si-EpCAM and 5-FU alone or together for 48 h, then stained with DAPI (400×). Condensed and fragmented nuclei in cells are indicated by arrowheads.

The effect of si-EpCAM and 5-FU on the morphology of MCF-7 cells was assessed by microscopic observation, which showed significant alterations in cell morphology in response to si-EpCAM in combination with 5-FU treatment for 48 h. MCF-7 cells exposed to 7.5 µg/ml and 20 µg/ml 5-FU began to shrink and floating cells appeared in the culture medium. Treatment with 7.5 µg/ml 5-FU combined with si-EpCAM resulted in an increased number of cells that lost contact with the surrounding cells and floating cells. In addition, the number of surviving cells decreased significantly when compared with the control group (Fig. 2A). Nuclear staining with DAPI revealed nuclear chromatin condensation in response to si-EpCAM and 5-FU alone or in combination, which is typical of apoptotic cells. In cells treated with 7.5 µg/ml 5-FU combined with si-EpCAM, the change was more obvious, while the cells in the control group showed diffuse uniform fluorescence (Fig. 2B). These results indicated that si-EpCAM in combination with 5-FU affected the morphology of MCF-7 cells and accelerated cell death.

### Knockdown of EpCAM in combination with 5FU increases apoptosis in breast cancer cells

**Figure 3:**
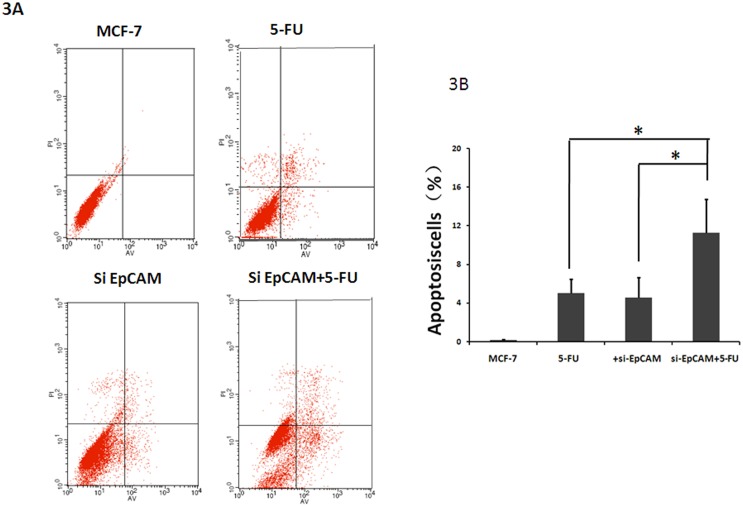
Effect of EpCAM silencing and/or 5-FU treatment on apoptosis in MCF-7 cells. A: Apoptosis was examined using annexin V-FITC/PI staining and flow cytometry analysis. A representative flow cytometric analysis of apoptosis in MCF-7 cells is shown. The fluorescence intensity of annexinV/FITC is plotted on the x-axis, and PI is plotted on the y-axis. FITC−/PI−, FITC+/PI−, FITC+/PI+, FITC−/PI+ was regarded as living, early apoptotic, late apoptotic and necrotic cells, respectively. B: The percentage of apoptotic cells was examined by annexin V-FITC/PI staining and flow cytometry analysis. Results are presented as mean±SD of three separate experiments. *P<0.05 versus 5-FU.

We investigated whether the effect of si-EpCAM and/or 5-FU treatment induced apoptosis using the Annexin V-FITC/PI staining method. To evaluate whether si-EpCAM promotes the chemosensitivity of 5-FU treated MCF-7 cells by up-regulating apoptosis, the apoptotic rate of MCF-7 cells was assessed following treatment with si-EpCAM and 5-FUalone or in combination for 48 h. The results showed that si-EpCAM in combination with 5-FUtreatment had a stronger effect on the induction of apoptosis than si-EpCAM and 5-FU alone (Fig. 3A and B). These finding suggested that si-EpCAM increased the chemosensitivity of 5-FU-treated MCF-7 cells by inducing apoptosis.

### Effects of EpCAM knockdown in combination with 5-FU on cell cycle progression

**Figure 4:**
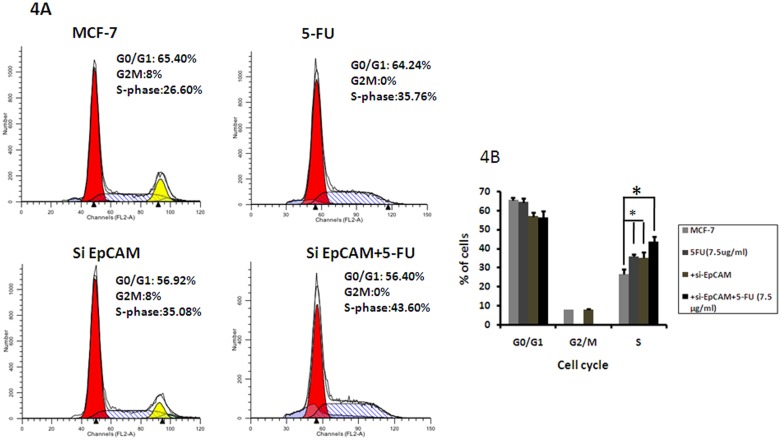
Effect of si-EpCAM and/or 5-FU treatment on cell cycle distribution in MCF-7 cells. Cells were incubated with si-EpCAM and 5-FU alone or in combination at the indicated concentrations for 48 h, and cell cycle distribution was evaluated using PI staining and flow cytometry. One representative flow cytometric analysis of cell cycle distribution is shown.

To examine whether the effect of EpCAM knockdown on chemosensitivity was related to the cell cycle, we examined cell cycle progression by flowcytometry. The cell cycle assay revealed that 5-FU alone and si-EpCAM alone increased the number of cells in S phase. Moreover, si-EpCAM in combination with 5-FU treatment caused more accumulation of cells in the S phase of the cell cycle than either treatment alone (5-FU: 35.76±2.01% vs.si-EpCAM+5-FU: 43.60±1.98%). The increase in the S-phase cell population was accompanied by a concomitant reduction of cells in G0/G1 and G2/M phases of cell cycle. Therefore, si-EpCAM in combination with 5-FU induced cell cycle arrest at the S phase more effectively than 5-FU alone (Fig. 4).

### Knockdown of EpCAM in combination with 5-FU promotes the chemosensitivity to 5-FU in breast cancer cells by downregulating the expression of Bcl-2

**Figure 5:**
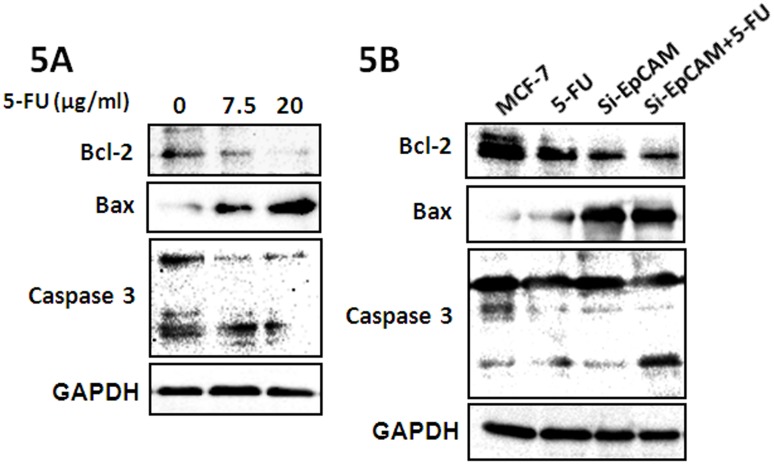
Effect of si-EpCAM and/or 5-FU treatment on apoptosis-related factors in MCF-7 cells. (A) MCF-7 cells were treated with 7.5 µg/ml and 20 µg/ml 5-FU for 48 h. Cells were harvested and analyzed by western blotting with antibodies against Bcl-2, Bax and caspase3. (B) MCF-7 cells were treated with si-EpCAM and/or 5-FU (7.5 µg/ml) for 48 h, and the expression of Bcl-2, Bax and caspase 3 was determined by immunoblotting.

The effect of si-EpCAM or 5-FU on the expression of Bcl-2 in MCF-7 cells was assessed by treating cells seeded in six-well plates with the indicated concentrations of 5-FU for 48 h, after which total protein was extracted and analyzed for the expression of apoptosis-related proteins. The results showed that 5-FU downregulated the expression of Bcl-2 and upregulated the expression of Bax and the levels of cleaved caspase3 in a dose-dependent manner (Fig. 5A). We further investigated the effect of si-EpCAM and/or 5-FU on apoptosis in MCF-7 cells. The results showed that si-EpCAM and 5-FU induced apoptosis in MCF-7 cells by downregulating the expression of the anti-apoptotic protein Bcl-2 and upregulating the expression of the pro-apoptotic proteins Bax, caspase3, and PARP and the effect was stronger when si-EpCAM and 5-FUwere used in combination (Fig. 5B). These results demonstrated that knockdown of EpCAM in combination with 5-FU may regulate cell apoptosis by modulating the expression of apoptosis-related proteins.

### Involvement of the ERK1/2 and JNK signaling pathways in si-EpCAM and 5-FU induced apoptosis

**Figure 6:**
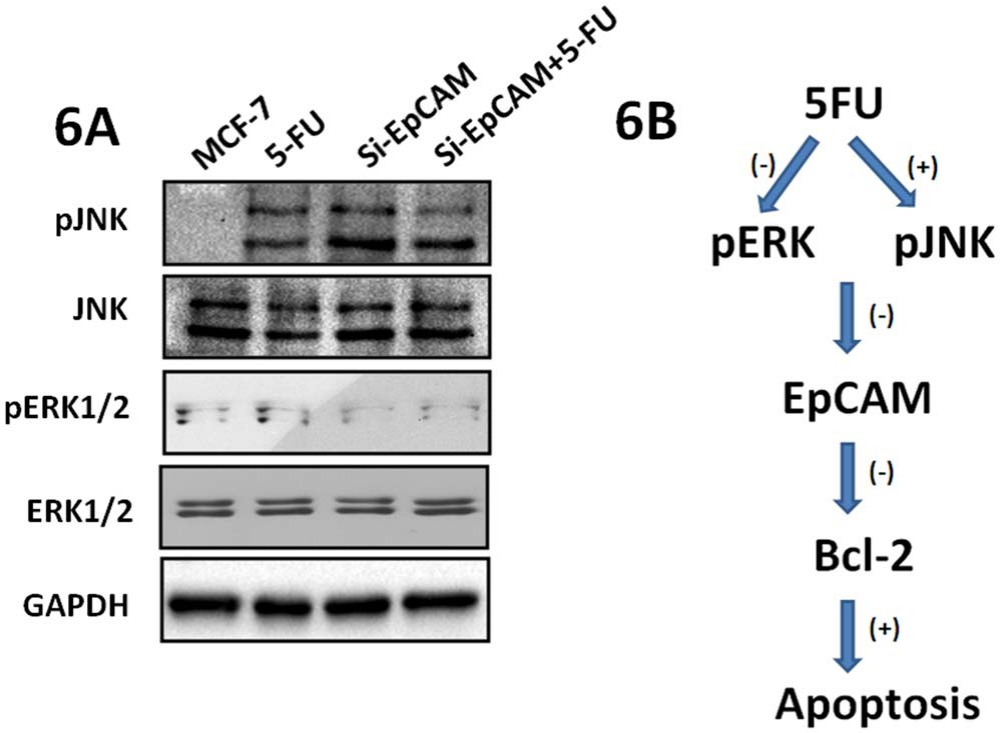
Involvement of the ERK1/2 and JNK signaling pathways in si-EpCAM and 5-FU induced apoptosis. (A) Effect of si-EpCAM and/or 5-FU treatment on the ERK and JNK signalingpathways.MCF-7 cells were treated with si-EpCAM and/or 5-FU (7.5 µg/ml) for 48 h, and the expression of pJNK and pERK1/2 was determined by immunoblotting. (B) The schematic illustration summarizes the effect of si-EpCAM on apoptosis in MCF-7 cells through activation of ERK and JNK signaling pathways induced by 5-FU.

Cell apoptosis is tightly controlled by a complex regulatory network. Since the JNK pathway is required for apoptosis induced by chemotherapeutic agents [16] and ERK1/2-mediated signaling plays a crucial role in mediating cell survival [17], it is conceivable that the JNK and ERK1/2pathways might also play a key role in si-EpCAM and 5-FU-induced apoptosis in MCF-7 cells. The expression of the p-JNK and pERK1/2 proteins was detectable in cells treated with si-EpCAM and/or 5-FU. 5-FU significantly increased the levels of pJNK inMCF-7 cells, and this effect was dramatically attenuated by pretreatment with si-EpCAM. In addition, 5-FU decreased the levels of pERK1/2, and this effect was also attenuated by pretreatment with si-EpCAM (Fig. 6A). These results supported the notion that both the ERK1/2 and JNK pathways contributed independently to si-EpCAM and 5-FU-induced apoptosis in MCF-7 cells. A schematic illustrating the relationship of all these apoptosis-related molecules and the effect of si-EpCAM and 5-FU on cell apoptosis is shown in Fig. 6B.

## Discussion

In the present study, we showed that knockdown of EpCAM significantly increased the chemosensitivity of MCF-7 cells to 5-FU in vitro through a mechanism involving the si-EpCAM-mediated modulation of the expression of anti-apoptotic factors in tumor cells and the induction of apoptosis and cell cycle arrest at S phase. This process was mediated by the ERK and JNK singling pathways. These findings provide new insight into the direct cytotoxic effect of EpCAM silencing and its application as a chemosensitizer.

EpCAM is overexpressed in many cancers including breast cancer and EpCAM overexpression is correlated with decreased survival of patients, making it an attractive diagnostic and therapeutic target in oncology [18], [19]. Previous studies reported that VB4-845, an immunotox in targeting EpCAM, showed potent cytotoxicity and was significantly effective in combination with 5-FU in hepatocellular carcinoma cells [20]. Treatment with an anti-EpCAM monoclonal antibody (MAb17-1A) combined with alpha-interferon; 5-FU and granulocyte-macrophage colony-stimulating factor could improve the therapeutic effect in patients with metastatic colorectal carcinoma [21]. These findings indicated that EpCAM-targeted therapy may offer a promising and novel approach for the treatment of cancer. However, the role of EpCAM in the chemosensitivity of breast cancer MCF-7 cells has not been investigated in vitro. The results of the present study demonstrated that knockdown of EpCAM could significantly increase the chemosensitivity of MCF-7 cells to 5-FU in vitro.

Apoptosis is the main mechanism of cell death induced by chemosensitizers. Recent studies showed that an immunotox in targeting EpCAM effectively inhibited peritoneal tumor growth in experimental models of mucinous peritoneal surface malignancies [22]. However, the effect of EpCAM on cell proliferation and apoptosis in breast cancer cells has not been assessed to date. We found that knockdown of EpCAM significantly inhibited the survival of MCF-7 cells (Fig. 1). Furthermore, we showed that apoptosis was responsible for si-EpCAM and/or 5FU induced cytotoxicity in MCF-7 cells using the CCK-8 assay, cell morphology assessment, DAPI staining and annexin V-PI staining (Figs. 1, 2, and 3). These results indicated that si-EpCAM increased the chemosensitivity to 5-FU in MCF-7 cells by increasing the rate of apoptosis.

Cell cycle arrest is another major mechanism of cell death induced by anti-tumor drugs [23], [24]. 5-FU is a fluoropyrimidine antimetabolite agent that is transformed inside the cell into different cytotoxic metabolites and is then incorporated into DNA and RNA, finally inducing cell cycle arrest and apoptosis by inhibiting the cell’s ability to synthesize DNA [25], [26]. 5-FU is widely used in the treatment of a variety of cancers including breast cancer [27]. In the present study, 7.5 µg/ml 5-FU induced cell cycle arrest at the S phase. Furthermore, we found that si-EpCAM in combination with 7.5 µg/ml 5-FU could further induce cells cycle arrest at the S phase compared with 5-FU alone (Fig. 4). These findings suggest that si-EpCAM in combination with 5-FU induced apoptosis by interrupting the transition of the cell cycle from S phase into G2/M phase, suggesting that the chemosensitizing effect of si-EpCAM was mediated by the induction of cell cycle arrest at S phase.

The proteins of the Bcl-2 family are key regulators of the mitochondrial pathway of apoptosis. Overexpression of the Bcl-2 protein is common in many human cancers, and contributes to their resistance to chemotherapy [28], [29], [30]. Bcl-2 overexpression in gastric cancer tumors was shown to predict the loss of efficacy of chemotherapies based on 5-FU, MMC or ADM [31]. The Bcl-2 gene, which is highly expressed in gallbladder carcinoma tissues, is one of the most important regulatory factors in cell apoptosis and plays an important role in the initiation and progression of gallbladder carcinoma [32]. The upregulation of Bcl-2 expression increases the resistance to chemotherapeutic drugs and radiotherapy, whereas the downregulation of Bcl-2 expression promotes the apoptotic response to anticancer drugs [33], [34]. Western blot experiments showed that si-EpCAM combined with 5-FU inhibited the expression of Bcl-2 in MCF-7 cells. These results suggested that the apoptosis induced by si-EpCAM and 5-FU is related to the downregulation of Bcl-2.

The mitogen-activated protein kinase (MAPK) cascades are evolutionary conserved, intracellular signal transduction pathways that respond to various extracellular stimuli and control a large number of fundamental cellular processes including growth, proliferation, differentiation, motility, stress response, survival and apoptosis. MAPKs include extracellular signal-regulated kinase (ERK), p38, and c-Jun NH2-terminal kinase (JNK) [35], [36]. The ERK signaling pathway plays a role in several steps of tumor development. JNK, which can be activated by various stimuli, induces multiple biological events and regulates cell death and survival. Previous studies have shown that JNK activation is related to cell survival, whereas prolonged JNK activation is associated with apoptotic cell death [37]. Therefore, we investigated whether knockdown of EpCAM enhanced apoptosis induced by 5-FU in MCF-7 cells. Our study showed that knockdown of EpCAM and 5-FUtreatment induced JNK activation and inhibited ERK1/2 activation in MCF-7 cells, which downregulated the expression of Bcl-2 and induced apoptosis.

In conclusion, our results showed that knockdown of EpCAM had a chemosensitizing effect by downregulating the expression of the anti-apoptotic factor Bcl-2 in MCF-7 cells, leading to the induction of apoptosis and cell cycle arrest at S phase when combined with 5-FU through a mechanism involving the modulation of the JNK and ERK1/2 signaling pathways. Therefore, EpCAM may be a potential candidate as a chemosensitizer in the management of breast cancer. It may provide a new idea for the clinical therapy.
